# Validation of the important role and prognostic value of KIF14 in triple-negative breast cancer

**DOI:** 10.1080/15384047.2025.2600705

**Published:** 2025-12-18

**Authors:** Jingjing Yuan, Meilin Zhang, Yaxuan Liu, Yiran Qiu, Mingdi Zhang, Hongliang Chen

**Affiliations:** a Department of Breast Surgery, Obstetrics & Gynecology Hospital of Fudan University, Shanghai Key Lab of Reproduction and Development, Shanghai Key Lab of Female Reproductive Endocrine Related Diseases, Shanghai, China

**Keywords:** KIF14, prognosis, TNBC, enrichment analysis, immune infiltration

## Abstract

**Background:**

Triple-negative breast cancer (TNBC) is an aggressive subtype with a poor prognosis and limited treatment options. Elevated Kinesin Family Member 14 (KIF14) expression in breast cancer (BC) is correlated with poor prognosis, but its role in TNBC remains unclear.

**Methods:**

KIF14 expression was analyzed using TCGA, TIMER, and GEO databases, and its association with prognosis was assessed via Kaplan‒Meier plotter. Functional assays, including CCK-8, wound healing, and Transwell assays, were performed to evaluate KIF14's impact on TNBC cell proliferation, migration, and invasion. GO and KEGG analyses of transcriptome data were used to explore molecular mechanisms. The relationship between KIF14 expression and immune infiltration was assessed in the TIMER database. KIF14 expression in clinical samples was validated using qRT-PCR and immunohistochemistry, and its correlation with clinical features was examined.

**Results:**

KIF14 was significantly upregulated in BC (*P* < 0.05), and elevated KIF14 expression was associated with poor prognosis. KIF14 knockdown reduced cell proliferation, migration, and invasion. Network analysis revealed its involvement in lipid metabolism, NF-κB, PI3K-AKT, and mTOR signaling pathways. Immune infiltration analysis showed a significant association between KIF14 and immune cell types.

**Conclusion:**

KIF14 promotes TNBC progression and serves as a potential diagnostic and prognostic biomarker for TNBC.

## Background

Breast cancer (BC) is the most common cancer and second leading cause of cancer-related deaths among the female population worldwide.^1^ The burden of breast cancer is not only reflected in its high incidence but also in the complexity of its diagnosis and treatment.^2^ One of the major challenges in breast cancer management is its heterogeneity, particularly in aggressive subtypes such as triple-negative breast cancer (TNBC), which is notoriously difficult to diagnose early and is associated with poor prognosis.^3^^,^^4^ Although several therapeutic approaches exist, including surgery, chemotherapy, targeted therapy, and immunotherapy, the development of drug resistance and relapse remains a significant obstacle.^5-7^ Moreover, the underlying mechanisms driving breast cancer initiation and progression are multifactorial, involving complex regulatory networks and signaling pathways. Despite decades of research, the precise molecular events and genetic alterations that contribute to breast cancer remain incompletely understood. Therefore, there is an urgent need for further investigation to uncover the intricacies of breast cancer biology, which could lead to more effective early detection methods and therapeutic strategies.

Kinesin family member 14 (KIF14) is a conserved, microtubule-dependent motor protein that plays a crucial role in various cellular processes.^8-10^ As a member of the kinesin superfamily, KIF14 exhibits ATPase activity and motor characteristics, allowing it to participate in key events of the cell cycle.^8^^,^^11^ These events include vesicle transport, mitotic spindle formation, chromosome segregation, and cytokinesis, all of which are essential for proper cell division and function.^12^ Emerging evidence suggests that KIF14 is overexpressed in several cancers, including prostate cancer, liver cancer, colorectal cancer, lung adenocarcinoma, cervical cancer, and ovarian cancer, positioning KIF14 as an oncogene with potential implications in cancer progression.^13-18^ This overexpression indicates that KIF14 may contribute to tumorigenesis by disrupting normal cellular division and facilitating the uncontrolled growth of cancer cells. Timothy W et al. reported that KIF14 expression was tumor-specific and increased in more aggressive tumors.^19^ Blood DNA samples from 105 breast cancer patients revealed KIF14 had prognostic value in breast cancer.^20^ An evaluation of 34 cases of locally advanced TNBC showed that KIF14 expression significantly correlated with chemotherapy-resistant breast cancer.^21^ However, the expression, prognostic significance, underlying mechanisms, and role of KIF14 in tumor immune regulation in TNBC remain poorly understood. In this study, we conducted bioinformatics analyses of public databases to examine the correlation between KIF14 expression and clinical characteristics in breast cancer. To further investigate the functional role of KIF14, we conducted in vitro experiments, which demonstrated that KIF14 promoted malignant behaviors on TNBC cells. Additionally, transcriptomic sequencing analysis revealed potential molecular mechanisms underlying KIF14’s role in TNBC. Moreover, immune infiltration analysis indicated that KIF14 exerts a multifaceted influence on the tumor microenvironment (TME). Further clinical sample analysis revealed KIF14's significant value as a potential biomarker in BC.

## Materials and methods

### BC patients and tissue microarray

Breast cancer patients who underwent surgical procedures between January 2021 and December 2022 at the Department of Breast Surgery, Obstetrics and Gynecology Hospital of Fudan University were included in this study. The tumor tissue microarrays (HBreD131Su08), purchased from Shanghai Biotech, contained 131 breast carcinoma samples with follow-up data ranging from 0 to 132 months. Written informed consent was obtained from all patients prior to their inclusion.

### Expression and prognostic analysis of KIF14 in BC

Pan-cancer analysis of KIF14 gene expression was conducted using data from The Cancer Genome Atlas (TCGA) (https://portal.gdc.cancer.gov/) and the GEO dataset (GSE205185). These databases were used to assess KIF14 mRNA expression levels and investigate the correlation between KIF14 expression and clinical features in breast cancer. The correlations between KIF14 and the overall survival (OS) and recurrence-free survival (RFS) were subsequently analyzed by the Kaplan‒Meier (KM) plotter database (http://kmplot.com/).^22^

### Transcriptome sequencing and bioinformatics analysis

We performed transcriptomic sequencing on the control and KIF14-knockdown cell lines for both cell types, and the resulting sequencing data were used for subsequent analysis. Genes exhibiting a similar downregulation pattern following KIF14 knockdown were analyzed using the GENEDENOVO online platform (https://www.omicsmart.com/#/). Genes with a log2(fold change) ≥ 2 were selected for heatmap visualization. We subsequently performed gene ontology (GO) analysis, including cellular component (CC), biological process (BP), and molecular function (MF) enrichment, as well as Kyoto encyclopedia of genes and genomes (KEGG) pathway analysis, on the 172 differentially expressed genes (FDR < 0.05, |log2(fold change) |>1) identified between the two groups.

### Tumor immune infiltration analysis

The tumor immune estimation resource (TIMER) (http://timer.cistrome.org/) is a comprehensive resource for analyzing tumor–immune interactions, encompassing 10,897 samples from 32 different cancer types.^23^ We utilized the correlation module of the TIMER database to explore the relationship between KIF14 expression and immune cell marker genes.

### Quantitative reverse transcription polymerase chain reaction (RT-qPCR)

Total RNA was extracted from the cell lines using the EZB RNA Extraction Kit (EZBioscience, USA). Reverse transcription was carried out with the Color Reverse Transcription Kit (EZBioscience, USA). For RT-qPCR, the 2× Color SYBR Green qPCR Master Mix (EZBioscience, USA) was used, following the manufacturer’s instructions. The RT-qPCR thermal cycling conditions were as follows: initial denaturation at 95 °C for 5 min, followed by 40 cycles of denaturation at 95 °C for 10 s and amplification at 60 °C for 30 s. Relative quantification was performed using the 2−ΔCT method. The relative mRNA levels were normalized against GAPDH. The primer sequences for PCR are shown in Table S1.

### Cell culture and cell transfection

Breast cancer cell lines (MCF-7, MDA-MB-231, MDA-MB-468, BT-549) were obtained from the cell bank of the Typical Culture Preservation Committee of the Chinese Academy of Sciences. The cells were cultured in RPMI-1640 or DMEM/high glucose medium, supplemented with 10% fetal bovine serum and 100 IU/mL penicillin-streptomycin at 37 °C in a 5% CO₂ incubator. Routine medium changes and passaging were performed. For the experiments, breast cancer cells in the logarithmic growth phase were harvested by digestion, counted, and plated. To establish stable KIF14-knockdown cell lines, MDA-MB-231 and BT-549 cells were transfected with lentiviral vectors. Stable clones were selected by treating cells with 2 µg/mL and 1 µg/mL puromycin for 4 weeks, respectively.

### Statistical analysis

Data processing and statistical analysis were conducted using GraphPad Prism 9 software, SPSS 27 and R version 4.3.2. Data analysis from the TCGA and GEO databases was performed using the appropriate R software packages. To compare KIF14 expression between two or more groups, the Kruskal‒Wallis or Wilcoxon tests were employed, depending on the data distribution. Logistic regression analysis was utilized to examine the association between KIF14 expression and clinical characteristics of BC. The prognostic significance of KIF14 was evaluated using Cox regression analysis. Continuous variables are presented as either mean± standard deviation or median with interquartile range (IQR), as appropriate. Differences between groups were assessed using the two-sample t-test or one-way ANOVA, based on the data type. The significance of categorical variables was determined using the chi-square test. Protein expression levels were analyzed using ImageJ software. All the statistical tests were two-sided, and a *P*-value of < 0.05 was considered statistically significant.

### Other methods

Additional methods are described in Appendix S1.

## Results

### KIF14 expression in breast cancer across databases

**Figure 1.**
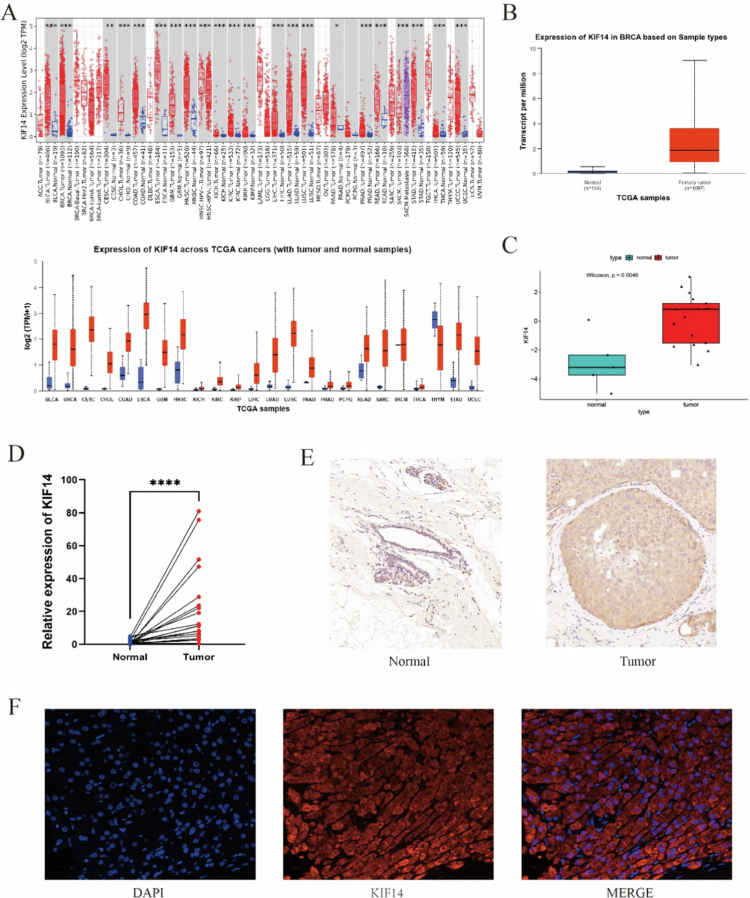
Expression of KIF14 in various tumors. (A) A pan-cancer analysis of KIF14 expression in the TIMER and TCGA databases. Note: red and blue represent tumor and normal samples, respectively. (B) KIF14 mRNA expression in BC tissues in the TCGA database. (C) KIF14 mRNA expression in BC tissues in the GEO database (GSE205185). (D) KIF14 mRNA expression in paired tumor and adjacent normal tissues from 20 patients in the hospital. (E) KIF14 protein expression was validated in BC and normal breast tissue by immunohistochemistry. (F) The localization of KIF14 protein in cells was demonstrated by immunofluorescence.

As shown in Figure 1a, analysis of the TIMER and ULCAN databases revealed that KIF14 expression was significantly upregulated in bladder, breast, cervical and other carcinomas compared to normal tissues (all *P* < 0.05). We further validated KIF14 mRNA expression in breast cancer (BC) using data from the TCGA database (Figure 1b). In line with these findings, the GSE205185 dataset also demonstrated elevated KIF14 mRNA expression in tumor tissues compared to normal tissues (Figure 1c). To corroborate these results, cancerous and adjacent tissues were collected from breast cancer patients at our hospital. Data analysis revealed significant upregulation of KIF14 at both the mRNA (Figure 1d) and protein (Figure 1e) levels in breast cancer tumor tissues. Immunofluorescence staining of tumor tissue samples with high KIF14 expression further confirmed that KIF14 was predominantly localized in both the cytoplasm and nucleus (Figure 1f).

### Association of KIF14 expression with prognosis in breast cancer

**Figure 2.**
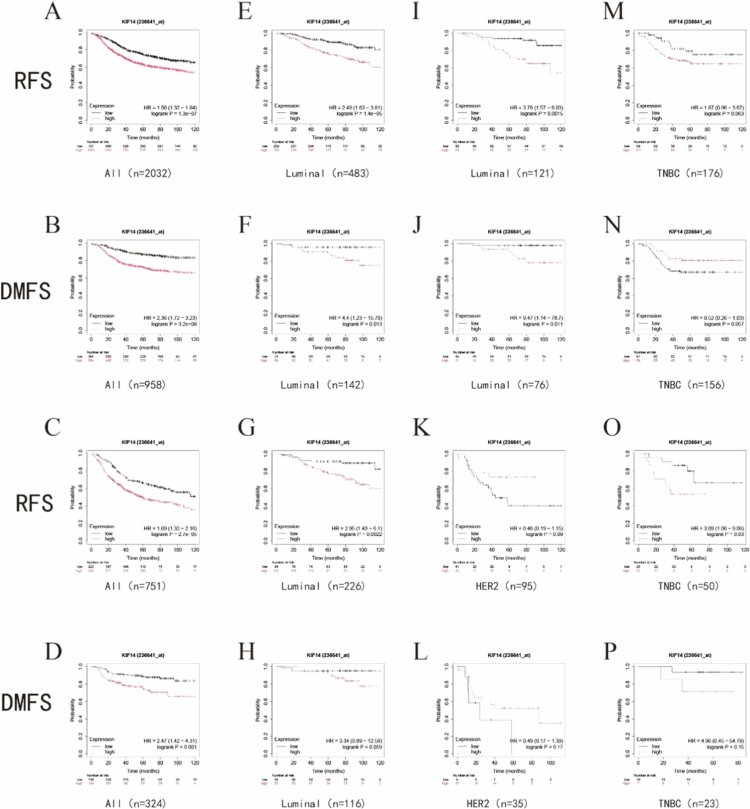
Survival analysis of BC patients stratified by median KIF14 expression. (A, B) Survival curves of RFS, DMFS in all breast cancers. (C, D) Survival curves of RFS, DMFS in all breast cancers (all patients received systemic treatment). (E, F) Survival curves of RFS, DMFS in luminal breast cancers. (G, H) Survival curves of RFS, DMFS in luminal breast cancers (all patients received endocrine therapy and any type of chemotherapy). (I, J) Survival curves of RFS, DMFS in luminal breast cancers (all patients received endocrine therapy but no chemotherapy). (K, L) Survival curves of RFS, DMFS in Her2+ enriched breast cancers. (M, N) Survival curves of RFS, DMFS in TNBC. (O, P) Survival curves of RFS, DMFS in TNBC (all patients received systemic treatment).

Figure 2 illustrates the correlation between KIF14 expression levels (cutoff value determined automatically by the system) and survival outcomes in BC using data from the KM Plotter database. High KIF14 expression was significantly associated with poor recurrence-free survival (RFS) and distant metastasis-free survival (DMFS) in all breast cancer subtypes (Figure 2a–d) as well as in luminal breast cancer (Figure 2e–j). However, the trend in HER2-enriched breast cancer (Figure 2k, l) and TNBC (Figure 2m–p) were less consistent. In HER2-enriched breast cancer, higher KIF14 expression was linked to longer RFS and DMFS, although these associations were not statistically significant (*P* > 0.05). Higher KIF14 expression in TNBC patients was associated with shorter RFS and longer DMFS, although no significant correlation was observed (Figure 2m and n). In TNBC patients who received systemic therapy, there was a substantial trend indicating that higher KIF14 expression was associated with shorter RFS, suggesting a higher likelihood of recurrence in the future (Figure 2o).

### KIF14 enhances proliferation, migration, and invasion in triple-negative breast cancer cells

**Figure 3.**
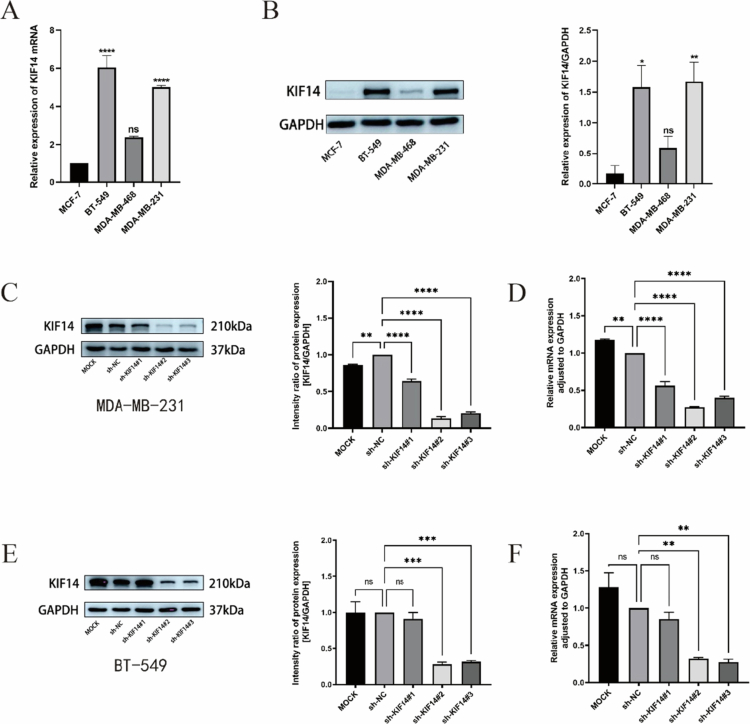
KIF14 expression in TNBC cells. (A, B) KIF14 mRNA (A) and protein (B) expression levels in four kinds of breast cancer cells. (C, D, E, F) The knockdown of KIF14 mRNA (D, F) and protein (C, E) expression were confirmed by qRT-PCR and Western blot. *P < 0.05, **P < 0.01, ***P < 0.001, ****P < 0.0001.

First, the expression levels of KIF14 were assessed in four breast cancer cell lines at both the mRNA (Figure 3a) and protein (Figure 3b) levels. qPCR and Western blot results showed that the relative levels of KIF14 expression was greater in highly invasive MDA-MB-231 and BT-549 cells than in less aggressive breast cancer cells. Given these findings, we selected MDA-MB-231 and BT-549 cells to knock down KIF14 using shRNA via lentiviral transfection. The efficiency of KIF14 silencing was validated at both the mRNA and protein levels (Figure 3c–f).

CCK-8 assays demonstrated that KIF14 knockdown significantly inhibited TNBC cell viability (Figure 4a). Colony formation assays further revealed a marked reduction in colony formation frequency in KIF14-silenced cells (Figure 4b). Additionally, wound healing assays showed that the migratory capacity of KIF14-knockdown cells was significantly impaired compared to the control group (Figure 4c). To assess the impact of KIF14 on cell invasion, transwell assays mimicking the cancer invasion process demonstrated a significant reduction in invasive ability in TNBC cells with KIF14 silencing (Figure 4d). Furthermore, flow cytometric analysis revealed that KIF14-depleted cells exhibited a greater propensity for arrest in the G0/G1 phase and a delay in progression through the S/G2 phases (Figure 4e).

Figure 4.KIF14 knockdown significantly inhibited TNBC cells proliferation, migration, and invasion in vitro. The effects of KIF14 on TNBC cells were evaluated based on growth curves (A) and colony formation assays (B). Wound healing assay (C) and transwell assay (D) were performed to examine KIF14-mediated cell invasion and migration. (E) The effect of KIF14 on the cell cycle was evaluated by flow cytometry. *P < 0.05, **P < 0.01, ***P < 0.001, ****P < 0.0001.

**Figure:**
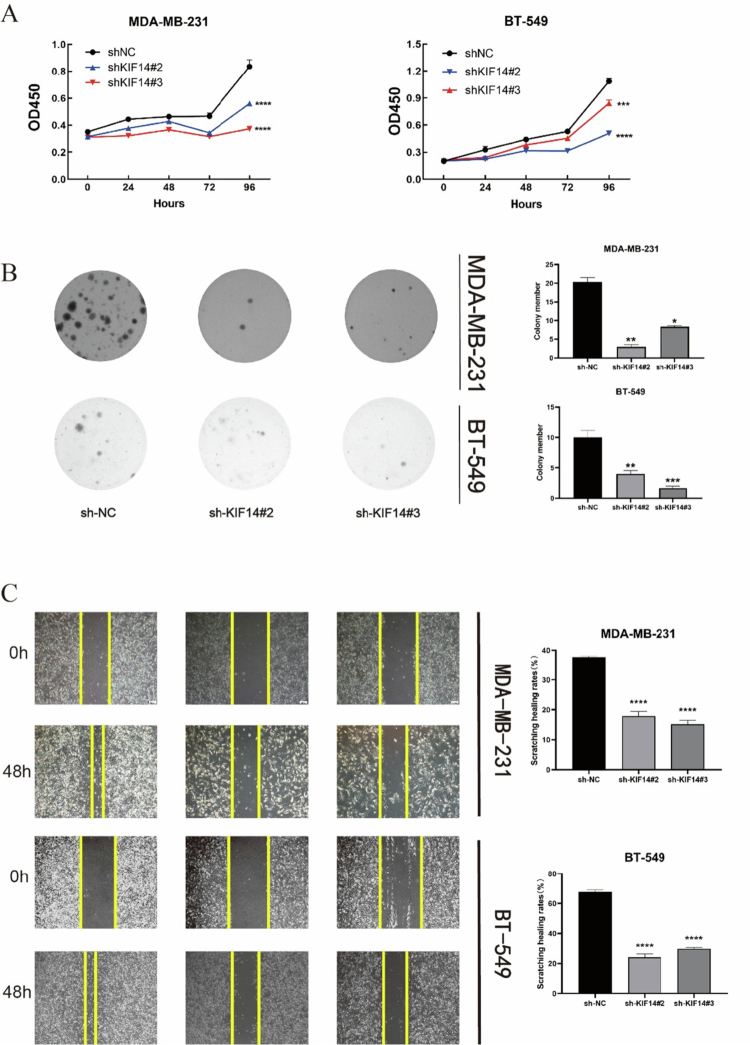

**Figure:**
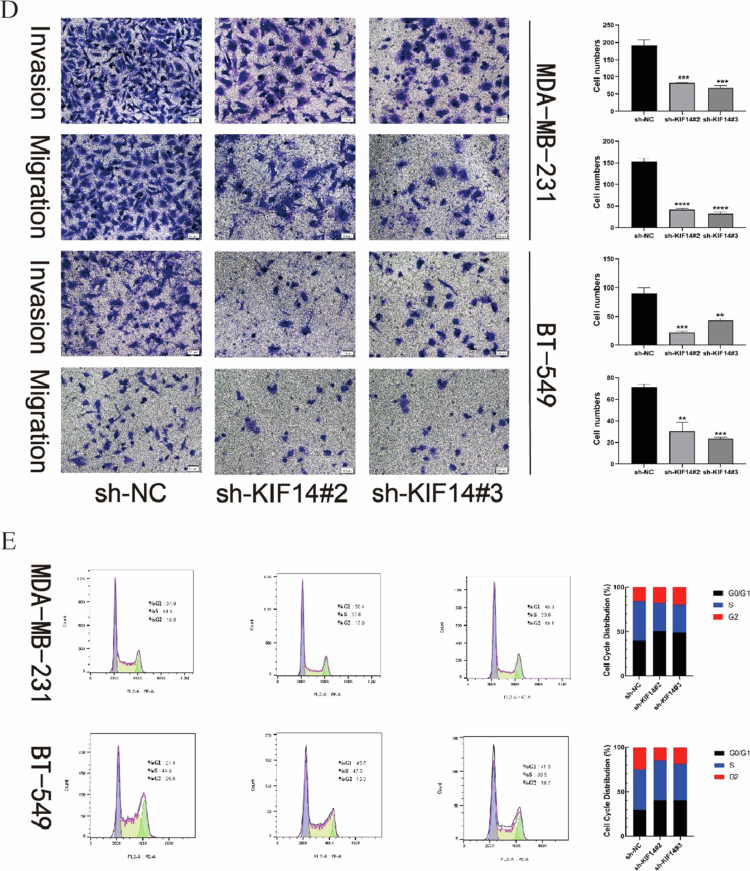

### Functional enrichment analysis of genes associated with KIF14 knockdown

**Figure 5.**
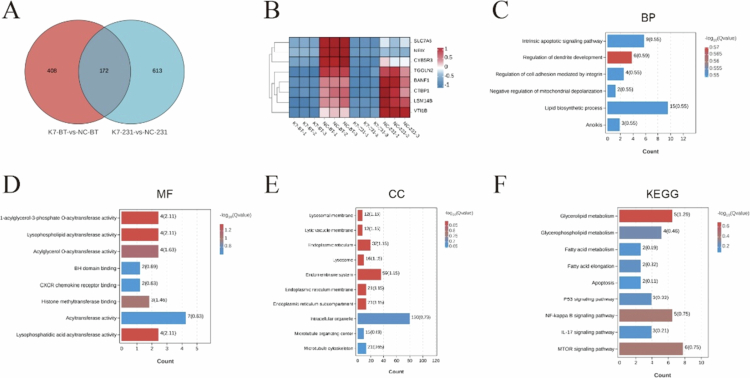
Functional enrichment analysis of genes associated with KIF14 knockdown. (A) Venn diagram shows the intersection of differentially expressed genes in two groups of cells. (B) Heat map shows negative genes associated with KIF14 in TNBC (top 8). Red represents an upregulated trend; blue represents a downregulated trend. (C) GO analysis (biological process) of 172 genes. (D) GO analysis (molecular functions) of 172 genes. (E) GO analysis (cellular components) of 172 genes. (F) KEGG analysis of 172 genes.

Transcriptome sequencing of KIF14-knockdown and control groups in MDA-MB-231 and BT-549 cells identified 580 and 785 differentially expressed genes, respectively. A total of 172 common genes were identified between the two cell lines (Figure 5a and b). GO and KEGG analyses revealed that KIF14-knockdown primarily affected pathways involved in microtubule structures, intracellular membranes, and lipid metabolism. Specifically, BP such as cell migration, apoptosis (anoikis pathway), and the stress response were significantly altered (Figure 5c). Additionally, MF related to lipid metabolism, epigenetic regulation, and signal transduction were notably impacted (Figure 5d). According to the CC analysis, genes related to lysosomal function, endoplasmic reticulum protein folding, and microtubule stabilization were downregulated (Figure 5e), potentially affecting tumor cell proliferation, metastasis, and drug resistance. KEGG pathway analysis highlighted the involvement of the NF-κB signaling pathway (Figure 5f).

### Correlation between KIF14 expression and tumor-infiltrating immune cells

**Figure 6.**
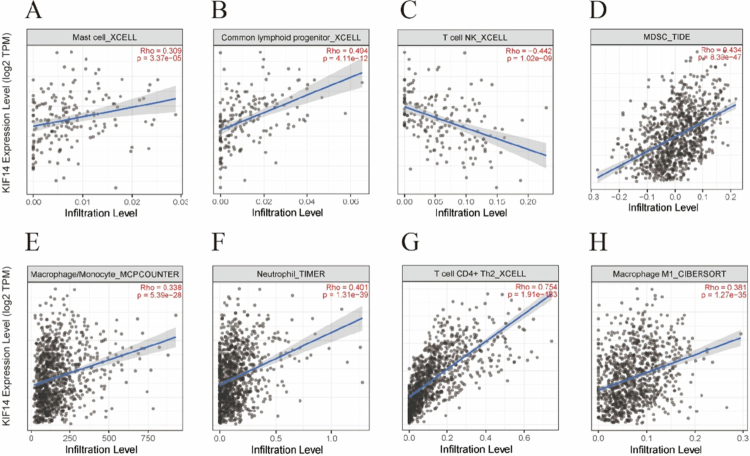
Relationship between KIF14 and immune cells in BC patients. Correlation between KIF14 expression and (A) mast cell, (B) common lymphoid progenitor, (C) T cell NK, (D) MDSC, (E) macrophage/monocyte, (F) neutrophil, (G) T cell CD4+ Th2, (H) macrophage M1.

**Figure 7.**
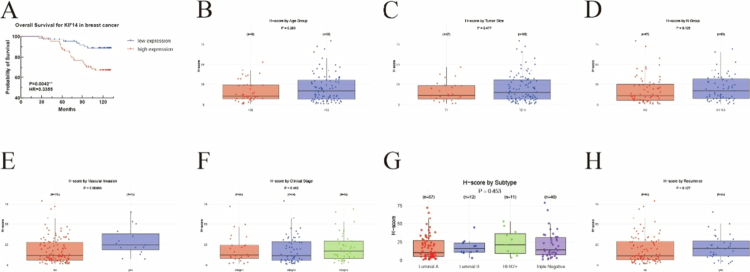
Correlation between KIF14 and the clinical features of BC patients. (A) Survival curves of OS in BC patients. KIF14 expression level in (B) age, (C) tumor size, (D) nodal metastasis, (E) vascular invasion, (F) clinical stages, (G) subtype, (H) recurrence.

To investigate the potential role of KIF14 in shaping the tumor immune microenvironment, we analyzed its correlation with the infiltration of various immune cell types using the TIMER database. As shown in Figure 6, KIF14 expression was positively correlated with several immune cell types, including mast cells (RHO = 0.309, *P* = 3.37 × 10^−5^) (Figure 6a), common lymphoid progenitors (CLPs) (RHO = 0.494, *P* = 4.11 × 10^−12^) (Figure 6b), T cell NK (Natural Killer T cells) (RHO = −0.442, *P* = 1.02 × 10^−9^) (Figure 6c), myeloid-derived suppressor cells (MDSCs) (RHO = 0.434, *P* = 8.39 × 10^−47^) (Figure 7d), macrophages/monocytes (RHO = 0.338, *P* = 5.39 × 10^−28^) (Figure 6e), neutrophils (RHO = 0.401, *P* = 1.31 × 10^−39^) (Figure 6f), and CD4+ Th2 T cells (RHO = 0.754, *P* = 1.91 × 10^−183^) (Figure 6g). The strongest positive correlation was observed in CD4+ Th2 T cells. Additionally, KIF14 expression was positively correlated with MDSCs, neutrophils, and macrophages/monocytes, suggesting a potential role in immune suppression and evasion. Conversely, a negative correlation with T cell NK was noted, indicating a distinct role for KIF14 in different immune cell subsets. These findings suggest that KIF14 may influence immune cell recruitment, polarization, and potentially immune evasion within the tumor microenvironment.

### Association of KIF14 expression with clinical outcomes and prognosis

To evaluate the clinical significance of KIF14 expression, immunohistochemical staining on tissue microarrays (TMAs) was performed to correlate KIF14 levels with OS, RFS, and clinicopathological features. The clinicopathologic characteristics were presented in Table S2. Moreover, the correlations between KIF14 levels and clinical factors in breast cancer patients were analyzed. Interestingly, patients with high KIF14 expression were more susceptible to tumor vascular invasion, had a higher pathological grade, and had a positive CD8 status (Table S2). In addition, to minimize the risk of omitting independent factors related to clinical characteristics, all factors with a univariate *p*-value < 0.1 were included in the multivariate analysis. Multivariate COX regression analysis showed that age (HR = 2.922, *P* = 0.036), nodal status (HR = 2.458, *P* = 0.020), TNBC status (HR = 2.879, *P* = 0.013), and KIF14 expression (HR = 2.650, *P* = 0.036) were independent risk factors for shorter OS (Table S3), but only nodal status (HR = 4.118, *P* < 0.001) was associated with shorter RFS (Table S4). As shown in Figure 7a, Kaplan‒Meier survival analysis revealed that high KIF14 expression was significantly associated with poor OS (HR = 3.28, *P* = 0.004), suggesting that KIF14 overexpression could serve as a prognostic marker. Figure 7b–h summarizes the results of univariate COX regression analysis across various clinical subgroups. Notably, KIF14 expression was significantly higher in tumors with vascular invasion (*P* < 0.05), suggesting a potential role in tumor vascularization. However, no significant associations were found between KIF14 expression and other clinical factors, including age, tumor size, lymph node stage, clinical stage, or subtype. These findings indicate that elevated KIF14 expression is associated with poor prognosis and may contribute to tumor progression, particularly in the context of vascular invasion.

## Discussion

KIF14, a member of the kinesin family of motor proteins, is a microtubule-dependent motor protein with ATPase activity and motor characteristics. KIF14 plays a crucial role in key processes during the cell cycle, including vesicle transport, spindle formation, chromosome segregation, and cytokinesis.^8-11^ It has been reported that KIF14 is involved in fetal neural and central developmental processes.^24^^,^^25^ When mutations in KIF14 cause abnormal cytoplasmic splitting, they lead to a range of disorders, such as microcephaly, intellectual disability, and renal dysplasia (RHD)-related syndromes, which may be useful for prenatal diagnosis.^26-28^ The elevated expression of KIF14 suggests that its expression and regulation are involved in multiple biological processes and pathologies beyond cancer. Recently, KIF14 has attracted attention in oncology, as it has been identified as a tumor-associated gene, with its overexpression linked to poor prognosis in several cancer types, including BC.^20^^,^^29-31^ In this study, we reported that KIF14 expression was significantly upregulated in breast cancer tissues compared to adjacent normal tissues, identified as an independent poor prognostic factor for BC. Functional assays demonstrated that KIF14 promotes TNBC cell proliferation, migration, and invasion, highlighting its role in facilitating tumor progression. Pathway enrichment analysis further demonstrated that KIF14 modulated critical intracellular signaling pathways, including the NF-κB, mTOR, and PI3K-Akt pathways, which played pivotal roles in TNBC cell survival and metastasis. Additionally, biological function enrichment analyses highlighted key processes such as cellular metabolism, stress response, and epigenetic regulation, all of which are significantly influenced by KIF14 in TNBC cells. Moreover, our findings suggested that KIF14 played a crucial role in modulating the tumor immune microenvironment, further complicating its involvement in cancer progression. Taken together, these results supported KIF14 as a potential oncogene in breast cancer (especially in TNBC), as both a diagnostic and prognostic biomarker. However, additional clinical validation is needed to fully establish its clinical utility in breast cancer management.

Previous studies have highlighted the critical role of KIF14 in tumorigenesis and its aberrant expression in various cancers. Brigitte et al. identified a cis-regulatory region containing Sp1, HSF1, and YY1 binding sites through promoter deletion analysis, which can be targeted by miRNA mimics and inhibitors to regulate KIF14 mRNA levels in ovarian cancer cell lines.^32^ Additionally, miRNAs such as miR-144-3p, miR-152, miR-17-3p, miR-340, and miR-154-5p have been shown to specifically target KIF14, providing potential therapeutic strategies for regulating its expression.^30^^,^^33-36^ In glioma, Xu et al. demonstrated that the long non-coding RNA PAXIP1-AS1 upregulates KIF14 expression by recruiting the transcription factor ETS1, promoting cell invasion and angiogenesis.^37^ Furthermore, Syed M. Ahmed et al. showed that KIF14 associates with the PDZ domain of Radil, negatively regulating Rap1-mediated integrin activation and inhibiting cell migration and invasion by binding Radil to microtubules.^38^ Several studies have also suggested a role for KIF14 in mediating chemoresistance in various cancers, although the precise mechanisms remain to be fully elucidated.^15^^,^^17^^,^^21^^,^^39^

Consistent with previous studies, our research demonstrated that KIF14 promoted BC growth and metastasis, as shown by bioinformatics analysis and in vitro experiments. To explore the downstream targets of KIF14 in BC pathogenesis, we identified key signaling pathways potentially regulated by KIF14, including lipid metabolism, NF-kappa B, PI3K-AKT, and mTOR. These pathways are known to play critical roles in tumor progression. For instance, KIF14 has been shown to enhance tumor cell proliferation and invasion through the AKT signaling pathway.^21^^,^^40^^,^^41^ In cholangiocarcinoma, KIF14 promotes tumor growth, lymphatic metastasis, and drug resistance via the NF-kappa B pathway.^31^ Furthermore, KIF14 has been implicated in regulating the cell cycle through the p27 pathway, thereby affecting cell cycle progression and tumor development.^42^^,^^43^ In summary, KIF14 likely promotes BC progression through a complex network of cancer-related signaling pathways.

Although only a few studies have examined the role of KIF14 in the tumor immune microenvironment, its involvement in immune regulation is gaining increasing attention.^16^ Previous research suggests that motor proteins like KIF14 may influence immune cell infiltration and tumor progression by modulating cellular signaling pathways. However, the precise contribution of KIF14 to the tumor immune landscape remains largely unexplored. In this study, we analyzed the correlation between KIF14 expression and the infiltration of various immune cell types in breast cancer. We found that KIF14 expression is positively correlated with the infiltration of CD4+ Th2 T cells, myeloid-derived suppressor cells (MDSCs), neutrophils, and macrophages/monocytes but negatively correlated with T cells and NK cells. These results suggested that KIF14 might exert differential effects on various immune cell subpopulations. CD4+ Th2 T cells, which are often linked to immune suppression and tumor progression,^44^ along with MDSCs and neutrophils, are known to promote tumor growth and metastasis by creating an immunosuppressive microenvironment.^45^^,^^46^ Macrophages, depending on their polarization (M1 or M2), can either support tumor progression or aid in immune defense,^47^ indicating that KIF14 might influence macrophage polarization. Conversely, the negative correlation with T cells and NK cells – key effectors of anti-tumor immunity – raises the possibility that KIF14 may be involved in immune evasion. These results suggested that KIF14 may play a complex role in regulating the recruitment, polarization, and potential immune escape of immune cells within the tumor microenvironment. Given these findings, further research is warranted to better understand how KIF14 modulates immune cell interactions and contributes to tumor immune evasion in breast cancer. Such insights could provide new avenues for therapeutic strategies targeting KIF14 to improve immune-based cancer treatments.

The data from KM Plotter revealed that survival outcomes in patients with TNBC and HER2-enriched subtypes were not consistent with those observed in the overall breast cancer cohort and luminal subtype patients, which may be attributable to the relatively small sample size in these groups. In contrast, the TMA analysis demonstrated a strong correlation between KIF14 expression and the TNBC subtype. Moreover, high KIF14 expression was associated with shorter OS and RFS.

There are several limitations in this study. First, all participants were recruited from a single-center, and future multi-center cohort studies are necessary to further strengthen these findings. Second, owing to the limited clinical follow-up data from the tissue microarrays, additional studies exploring the correlation with other factors are not possible. Furthermore, the specific relationship between KIF14 and the tumor immune microenvironment, as well as the underlying mechanisms, requires further experimental validation. Next, while this study revealed that KIF14 may affect signaling pathways related to lipid metabolism and tumor malignancy, its direct or indirect relationship has not been experimentally validated. Finally, whether KIF14 also contributes to the development of other subtypes of breast cancer remains an area for future investigation.

## Conclusion

In conclusion, KIF14 expression is markedly elevated in breast cancer, and its high expression correlates with poorer prognosis, highlighting its potential as both a diagnostic and a prognostic biomarker in breast cancer. Moreover, our findings suggest that KIF14 may play a role in modulating immune cell infiltration within the tumor microenvironment. These insights provide a strong theoretical basis for the development of novel, non-invasive diagnostic approaches for breast cancer. However, further investigations are crucial to fully elucidate the underlying mechanisms and validate the clinical utility of KIF14 in breast cancer management.

## Consent for publication

All the authors have read and agreed to the published version of the manuscript.

## Supplementary Material

Supplementary material Additional methods

Supplementary Material Supplementary_Tables_CLEAN_COPY.docx

## Data Availability

All the data produced or examined throughout this study are contained within this manuscript and its supplementary information files. Additional data supporting the findings of this study are available from the corresponding author on reasonable request.

## References

[1] Xu J, Gao F, Liu W, Guan X. Cell-cell communication characteristics in breast cancer metastasis. Cell Commun Signaling. 2024;22(1):55. doi: 10.1186/s12964-023-01418-4. PMC10799417 38243240

[2] Zhang Y, Gong S, Liu X. Spatial transcriptomics: a new frontier in accurate localization of breast cancer diagnosis and treatment. Front Immunol. 2024;15:1483595. doi: 10.3389/fimmu.2024.1483595. 39439806 PMC11493667

[3] Jovanović B, Temko D, Stevens LE, Seehawer M, Fassl A, Murphy K, Anand J, Garza K, Gulvady A, Qiu X, et al. Heterogeneity and transcriptional drivers of triple-negative breast cancer. Cell Rep. 2023;42(12):113564. doi: 10.1016/j.celrep.2023.113564. 38100350 PMC10842760

[4] Anderle N, Schäfer-Ruoff F, Staebler A, Kersten N, Koch A, Önder C, Keller A, Liebscher S, Hartkopf A, Hahn M, et al. Breast cancer patient-derived microtumors resemble tumor heterogeneity and enable protein-based stratification and functional validation of individualized drug treatment. J Exp Clin Cancer Res. 2023;42(1):210. doi: 10.1186/s13046-023-02782-2. 37596623 PMC10436441

[5] Kerr AJ, Dodwell D, McGale P, Holt F, Duane F, Mannu G, Darby SC, Taylor CW. Adjuvant and neoadjuvant breast cancer treatments: a systematic review of their effects on mortality. Cancer Treat Rev. 2022;105:102375. doi: 10.1016/j.ctrv.2022.102375. 35367784 PMC9096622

[6] Sun L, Jia X, Wang K, Li M. Unveiling the future of breast cancer therapy: cutting-edge antibody-drug conjugate strategies and clinical outcomes. Breast. Dec 2024;78:103830. doi: 10.1016/j.breast.2024.103830. 39500221 PMC11570738

[7] Barzaman K, Karami J, Zarei Z, Hosseinzadeh A, Kazemi MH, Moradi-Kalbolandi S, Safari E, Farahmand L. Breast cancer: biology, biomarkers, and treatments. Int Immunopharmacol. 2020;84:106535. doi: 10.1016/j.intimp.2020.106535. 32361569

[8] Benoit M, Asenjo AB, Paydar M, Dhakal S, Kwok BH, Sosa H. Structural basis of mechano-chemical coupling by the mitotic kinesin KIF14. Nat Commun. 021;12(1):3637. doi: 10.1038/s41467-021-23581-3. 34131133 PMC8206134

[9] Carleton M, Mao M, Biery M, Warrener P, Kim S, Buser C, Marshall CG, Fernandes C, Annis J, Linsley PS. RNA interference-mediated silencing of mitotic kinesin KIF14 disrupts cell cycle progression and induces cytokinesis failure. Mol Cell Biol. 2006;26(10):3853–3863. doi: 10.1128/mcb.26.10.3853-3863.2006. 16648480 PMC1488988

[10] Yang Z, Li C, Yan C, Liu B, Zhu Z, Wu Y, Gu Q. KIF14 promotes tumor progression and metastasis and is an independent predictor of poor prognosis in human gastric cancer. Biochim Biophys Acta Mol Basis Dis. 2019;1865(1):181–192. doi: 10.1016/j.bbadis.2018.10.039. 30404039

[11] Miki H, Setou M, Kaneshiro K, Hirokawa N. All kinesin superfamily protein, KIF, genes in mouse and human. Proc Natl Acad Sci U S A. 2001;98(13):7004–7011. doi: 10.1073/pnas.111145398. 11416179 PMC34614

[12] Hirokawa N. Kinesin and dynein superfamily proteins and the mechanism of organelle transport. Science. 1998;279(5350):519–526. doi: 10.1126/science.279.5350.519. 9438838

[13] Liu L, Li M, Zhang J, Xu D, Guo Y, Cang S. KIF14 mediates cabazitaxel-docetaxel cross-resistance in advanced prostate cancer by promoting AKT phosphorylation. Arch Biochem Biophys. 2023;737 109551. 10.1016/j.abb.2023.109551. 36822388

[14] Yu R, Wu X, Qian F, Yang Q. RFC3 drives the proliferation, migration, invasion and angiogenesis of colorectal cancer cells by binding KIF14. Exp Ther Med. 2024;27(5):222. doi: 10.3892/etm.2024.12510. 38590579 PMC11000453

[15] Cheng C, Wu X, Shen Y, Li Q. KIF14 and KIF23 promote cell proliferation and chemoresistance in HCC cells, and predict worse prognosis of patients with HCC. Cancer Manag Res. 2020;12:13241–13257. doi: 10.2147/cmar.S285367. 33380832 PMC7767722

[16] Wang H, Tang F, Tang P, Zhang L, Gan Q, Li Y. Noncoding RNAs-mediated overexpression of KIF14 is associated with tumor immune infiltration and unfavorable prognosis in lung adenocarcinoma. Aging. 2022;14(19):8013–8031. doi: 10.18632/aging.204332. 36227151 PMC9596199

[17] Wang W, Shi Y, Li J, Cui W, Yang B. Up-regulation of KIF14 is a predictor of poor survival and a novel prognostic biomarker of chemoresistance to paclitaxel treatment in cervical cancer. Biosci Rep. 2016;36(2), 10.1042/bsr20150314. PMC4820787 27128470

[18] Qiu HL, Deng SZ, Li C, Tian ZN, Song XQ, Yao GD, Geng JS. High expression of KIF14 is associated with poor prognosis in patients with epithelial ovarian cancer. Eur Rev Med Pharmacol Sci. 2017;21(2):239–245. 28165566

[19] Corson TW, Gallie BL. KIF14 mRNA expression is a predictor of grade and outcome in breast cancer. Int J Cancer. 2006;119(5):1088–1094. doi: 10.1002/ijc.21954. 16570270

[20] Krus I, Brynychová V, Hlaváč V, Václavíková R, Kováčová M, Koževnikovová R, Kopečková K, Tornikidis J, Vrána D, Gatěk J, et al. Single nucleotide variants in KIF14 gene may have prognostic value in breast cancer. Mol Diagn Ther. 2022;26(6):665–678. doi: 10.1007/s40291-022-00616-z. 36192583

[21] Singel SM, Cornelius C, Zaganjor E, Batten K, Sarode VR, Buckley DL, Peng Y, John GB, Li HC, Sadeghi N, et al. KIF14 promotes AKT phosphorylation and contributes to chemoresistance in triple-negative breast cancer. Neoplasia. 2014;16(3):247–256.e2. doi: 10.1016/j.neo.2014.03.008. 56. 24784001 PMC4094827

[22] Lánczky A, Győrffy B. Web-based survival analysis tool tailored for medical research (KMplot): development and implementation. J Med Internet Res. 2021;23(7):e27633. doi: 10.2196/27633. 34309564 PMC8367126

[23] Li T, Fan J, Wang B, Traugh N, Chen Q, Liu JS. TIMER: a web server for comprehensive analysis of tumor-infiltrating immune cells. Cancer Res. 2017;77(21):e108–e110. doi: 10.1158/0008-5472.Can-17-0307. 29092952 PMC6042652

[24] Makrythanasis P, Maroofian R, Stray-Pedersen A, Musaev D, Zaki MS, Mahmoud IG, Selim L, Elbadawy A, Jhangiani SN, Coban Akdemir ZH, et al. Biallelic variants in KIF14 cause intellectual disability with microcephaly. Eur J Hum Genet. 2018;26(3):330–339. doi: 10.1038/s41431-017-0088-9. 29343805 PMC5839044

[25] Fujikura K, Setsu T, Tanigaki K, Abe T, Kiyonari H, Terashima T, Sakisaka T, Smeyne RJ. Kif14 mutation causes severe brain malformation and hypomyelination. PLoS One. 2013;8(1):e53490. doi: 10.1371/journal.pone.0053490. 23308235 PMC3537622

[26] Filges I, Nosova E, Bruder E, Tercanli S, Townsend K, Gibson W, Röthlisberger B, Heinimann K, Hall J, Gregory‐Evans C, et al. Exome sequencing identifies mutations in KIF14 as a novel cause of an autosomal recessive lethal fetal ciliopathy phenotype. Clin Genet. 2014;86(3):220–228. doi: 10.1111/cge.12301. 24128419

[27] Reilly ML, Stokman MF, Magry V, Jeanpierre C, Alves M, Paydar M, Hellinga J, Delous M, Pouly D, Failler M, et al. Loss-of-function mutations in KIF14 cause severe microcephaly and kidney development defects in humans and zebrafish. Hum Mol Genet. 2019;28(5):778–795. doi: 10.1093/hmg/ddy381. 30388224 PMC6381319

[28] Moawia A, Shaheen R, Rasool S, Waseem SS, Ewida N, Budde B, Kawalia A, Motameny S, Khan K, Fatima A, et al. Mutations of KIF14 cause primary microcephaly by impairing cytokinesis. Ann Neurol. 2017;82(4):562–577. doi: 10.1002/ana.25044. 28892560

[29] Jin T, Ding L, Chen J, Zou X, Xu T, Xuan Z, Wang S, Zhu C, Zhang Y, Huang P, et al. BUB1/KIF14 complex promotes anaplastic thyroid carcinoma progression by inducing chromosome instability. J Cell Mol Med. 2024;28(7):e18182. doi: 10.1111/jcmm.18182. 38498903 PMC10948175

[30] Li Y, Hong X, Zhai J, Liu Y, Wang X, Zhang Y, Lv Q. Novel circular RNA circ-0002727 regulates miR-144-3p/KIF14 pathway to promote lung adenocarcinoma progression. Front Cell Dev Biol. 2023;11:1249174. doi: 10.3389/fcell.2023.1249174. 38033864 PMC10686231

[31] Jiang W, Wang J, Yang X, Shan J, Zhang Y, Shi X, Chenyan A, Chang J, Yu Y, Li C. KIF14 promotes proliferation, lymphatic metastasis and chemoresistance through G3BP1/YBX1 mediated NF-κB pathway in cholangiocarcinoma. Oncogene. 2023;42(17):1392–1404. doi: 10.1038/s41388-023-02661-2. 36922675

[32] Thériault BL, Basavarajappa HD, Lim H, Pajovic S, Gallie BL, Corson TW. Transcriptional and epigenetic regulation of KIF14 overexpression in ovarian cancer. PLoS One. 2014;9(3):e91540. doi: 10.1371/journal.pone.0091540. 24626475 PMC3953446

[33] Meng F, Zhang Z. MicroRNA-152 specifically targets kinesin family member 14 to suppress the advancement of bladder cancer cells via PI3K/AKT pathway. Biochem Biophys Res Commun. 2024;692:149337. doi: 10.1016/j.bbrc.2023.149337. 38070277

[34] Ji J, Fu J. MiR-17-3p facilitates aggressive cell phenotypes in colon cancer by targeting PLCD1 through affecting KIF14. Appl Biochem Biotechnol. 2023;195(3):1723–1735. doi: 10.1007/s12010-022-04218-7. 36367621

[35] Xu HK, Wang XD, Wang DG, Wei DD, Liang L, Liu CH. miR-340 exerts suppressive effect on retinoblastoma progression by targeting KIF14. Curr Eye Res. 2021;46(2):232–238. doi: 10.1080/02713683.2020.1795202. 32757684

[36] Chen J, Ma C, Zhang Y, Pei S, Du M, Qian L, Wang J, Yin L, He X. MiR-154-5p suppresses cell invasion and migration through inhibiting KIF14 in nasopharyngeal carcinoma. Onco Targets Ther. 2020;13:2235–2246. doi: 10.2147/ott.S242939. 32214824 PMC7078655

[37] Xu H, Zhao G, Zhang Y, Jiang H, Wang W, Yu H, Qi L. Long non-coding RNA PAXIP1-AS1 facilitates cell invasion and angiogenesis of glioma by recruiting transcription factor ETS1 to upregulate KIF14 expression. J Exp Clin Cancer Res. 2019;38(1):486. doi: 10.1186/s13046-019-1474-7. 31823805 PMC6902534

[38] Ahmed SM, Thériault BL, Uppalapati M, Chiu CW, Gallie BL, Sidhu SS, Angers S. KIF14 negatively regulates Rap1a-Radil signaling during breast cancer progression. J Cell Biol. 2012;199(6):951–967. doi: 10.1083/jcb.201206051. 23209302 PMC3518219

[39] Singel SM, Cornelius C, Batten K, Fasciani G, Wright WE, Lum L, Shay JW. A targeted RNAi screen of the breast cancer genome identifies KIF14 and TLN1 as genes that modulate docetaxel chemosensitivity in triple-negative breast cancer. Clin Cancer Res. 2013;19(8):2061–2070. doi: 10.1158/1078-0432.Ccr-13-0082. 23479679 PMC4513911

[40] Liu L, Li M, Zhang J, Xu D, Guo Y, Cang S. KIF14 mediates cabazitaxel-docetaxel cross-resistance in advanced prostate cancer by promoting AKT phosphorylation. Arch Biochem Biophys. 2023;737:109551. doi: 10.1016/j.abb.2023.109551. 36822388

[41] Wang ZZ, Yang J, Jiang BH, Di J, Gao P, Peng L, Su X. KIF14 promotes cell proliferation via activation of Akt and is directly targeted by miR-200c in colorectal cancer. Int J Oncol. 2018;53(5):1939–1952. doi: 10.3892/ijo.2018.4546. 30226594 PMC6192758

[42] Zhang J, Buranjiang G, Mutalifu Z, Jin H, Yao L. KIF14 affects cell cycle arrest and cell viability in cervical cancer by regulating the p27(Kip1) pathway. World J Surg Oncol. 2022;20(1):125. doi: 10.1186/s12957-022-02585-3. 35439960 PMC9016959

[43] Xu H, Choe C, Shin SH, Park S, Kim H, Jung S, Yim S, Chung Y. Silencing of KIF14 interferes with cell cycle progression and cytokinesis by blocking the p27(Kip1) ubiquitination pathway in hepatocellular carcinoma. Exp Mol Med. 2014;46(5):e97–e97. doi: 10.1038/emm.2014.23. 24854087 PMC4044675

[44] Chen Y, Teng Y, Xu P, Wang S. The role of citrullination modification in CD4(+) T cells in the pathogenesis of immune-related diseases. Biomolecules. 2024;14(4):400. doi: 10.3390/biom14040400. 38672418 PMC11047979

[45] Goldmann O, Medina E. Metabolic pathways fueling the suppressive activity of myeloid-derived suppressor cells. Front Immunol. 2024;15:1461455. doi: 10.3389/fimmu.2024.1461455. 39534601 PMC11554506

[46] Herro R, Grimes HL. The diverse roles of neutrophils from protection to pathogenesis. Nat Immunol. Dec 2024;25(12):2209–2219. doi: 10.1038/s41590-024-02006-5. 39567761

[47] Murrey MW, Ng IT, Pixley FJ. The role of macrophage migratory behavior in development, homeostasis and tumor invasion. Front Immunol. 2024;15:1480084. doi: 10.3389/fimmu.2024.1480084. 39588367 PMC11586339

