# Supplementary material for: Validation of the important role and prognostic value of KIF14 in triple-negative breast cancer
Source: Cancer Biol Ther. 2025 Dec 18;26(1):2600705. doi: 10.1080/15384047.2025.2600705 (PMC12919895; doi:10.1080/15384047.2025.2600705)
Supplement: Supplementary material — Additional methods [file KCBT_A_2600705_SM2317.docx]

**Appendix S1**

**Immunohistochemistry**

Immunohistochemistry was performed using the avidin-biotin complex method (Vector Laboratories), with heat-induced antigen retrieval. The slides were incubated with antibodies against KIF14 (1:200; ab71155, Abcam). KIF14 expression was quantified using 3D HISTECH Quant Center 2.0 analysis software. Immunostaining was assessed using a quantitative imaging approach, recording both the percentage of positive cells and staining intensity. The H-score was calculated according to the formula: H-score = Σ(PI × I) = (percentage of cells with weak intensity × 1) + (percentage of cells with moderate intensity × 2) + (percentage of cells with strong intensity × 3), where PI represents the percentage of positive cells relative to all cells, and I corresponds to the staining intensity.

**Western blotting**

Total cell lysates were prepared and separated by sodium dodecyl sulfate-polyacrylamide gel electrophoresis (SDS-PAGE), then transferred to a PVDF membrane. The membrane was blocked with 5% skimmed milk for 1 hour at room temperature, followed by overnight incubation at 4°C with primary antibodies against KIF14 (1:200; ab71155, Abcam) and GAPDH (1:10,000; ab8245, Abcam). The membrane was then incubated with a goat anti-rabbit IgG HRP-conjugated secondary antibody (Proteintech, Cat No: SA00001-2, 1:10,000) for 1 hour at room temperature. Protein signals were detected using the High ECL Assay Reagent (Ncmbio, Cat No. P10060, Suzhou, China) according to the manufacturer's protocol. Images were captured using a Tanon imaging system.

**Colony formation assay and CCK-8 assay**

Different cell lines were inoculated into 6-well plates and cultured in the appropriate medium at 37°C, with the cell density adjusted accordingly. Colonies were counted after 15 days of culture. Approximately 1,000 cells per well were seeded in 96-well plates and treated with 100 μL of medium including 10 μL of CCK-8 reagent (Beyotime). After incubation for 2 h at 37°C with 5% CO2, the optical density of each well was measured at 450 nm by an enzyme marker (Bio-tek). All experiments were performed in triplicate.

**Wound healing assay**

Transfected breast cancer cells were cultured in six-well plates until they reached confluence. A sterile 100 μL pipette tip was used to create straight lines across the monolayer, generating cell-free areas. The cells were photographed at 0, 24, and 48 hours. Images were captured from five random fields of view for each sample, and each experiment was performed in triplicate.

**Transwell assay**

The transwell chambers were pre-coated with Matrigel. Transfected breast cancer cells were resuspended in serum-free medium, and 200 μL of the cell suspension (5,000 cells/well for MDA-MB-231 cells; 100,000 cells/well for BT-549 cells) was added to the upper chamber. In the lower chamber, 600 μL of medium containing 10% fetal bovine serum was added. After 24 hours, the transwell chamber was removed; firstly, it was fixed with 4% paraformaldehyde for 30 minutes, and secondly, it was stained with crystal violet for 20 minutes. Images were captured following random microscopic observations.
