# Supplementary material for: Validation of the important role and prognostic value of KIF14 in triple-negative breast cancer
Source: Cancer Biol Ther. 2025 Dec 18;26(1):2600705. doi: 10.1080/15384047.2025.2600705 (PMC12919895; doi:10.1080/15384047.2025.2600705)
Supplement: Supplementary Material — Supplementary_Tables_CLEAN_COPY.docx [file KCBT_A_2600705_SM8068.docx]

**Table S1: Primer sequences used in qRT-PCR.**

| Primer name | Forward | Reverse |
| --- | --- | --- |
| KIF14 | 5'–TGTAGGTAGATTGGCACTTCAGA–3' | 5'–CGACGTTGTAATGTAAGACGTGT–3' |
| GAPDH | 5'–CAGGAGGCATTGCTGATGAT–3' | 5'–GAAGGCTGGGGCTCATTT–3' |

**Table S2.** Association of KIF14 expression with the clinicopathological features of 131 breast cancer patients.

|  |  | | KIF14 expression | |  |
| --- | --- | --- | --- | --- | --- |
|  | n (%) | | Low (%) | High (%) | P value |
| **Age（years）** |  | |  |  | 0.168 |
| <50 | 41 (31.3) | | 24 (58.5) | 17 (41.5) |  |
| ≥50 | 90 (68.7) | | 41 (45.6) | 49 (54.4) |  |
| **Tumor size（cm）** |  | |  |  | 0.544 |
| ≤2 | 61 (46.6) | | 32 (52.5) | 29 (47.5) |  |
| ＞2 | 70 (53.4) | | 33 (47.1) | 37 (52.9) |  |
| **Nodal status** |  | |  |  | 0.392 |
| N0-1 | 86 (65.6) | | 45 (52.3) | 41 (47.7) |  |
| N2-3 | 45 (34.4) | | 20 (44.4) | 25 (55.6) |  |
| **Clinical TNM stages** | |  |  |  | 0.609 |
| 1 | 36 (27.5) | | 20 (55.6) | 16 (44.4) |  |
| 2 | 50 (38.2) | | 25 (50.0) | 25 (50.0) |  |
| 3 | 45 (34.3) | | 20 (44.4) | 25 (55.6) |  |
| **Pathological grade** |  | |  |  | 0.017* |
| Ⅱ | 90 (68.7) | | 51 (56.7) | 39 (43.3) |  |
| Ⅲ | 41 (31.3) | | 14 (34.1) | 27 (65.9) |  |
| **PD-L1 status** |  | |  |  | 0.037* |
| Negative | 45 (34.4) | | 28 (62.2) | 17 (37.8) |  |
| Positive | 86 (65.6) | | 37 (43.0) | 49 (57.0) |  |
| **CD8 status** |  | |  |  | 0.571 |
| Negative | 115 (87.8) | | 56 (48.7) | 59 (51.3) |  |
| Positive | 16 (12.2) | | 9 (56.3) | 7 (43.7) |  |
| **Vascular invasion** |  | |  |  | 0.007* |
| No | 116 (88.5) | | 63 (54.3) | 53 (45.7) |  |
| Yes | 15 (11.5) | | 2 (13.3) | 13 (86.7) |  |
| **Molecular subtype** |  | |  |  | 0.77 |
| Luminal A | 67 (51.1) | | 36 (53.7) | 31 (46.3) |  |
| Luminal B | 12 (9.2) | | 6 (50.0) | 6 (50.0) |  |
| HER2 enriched | 11 (8.4) | | 5 (45.5) | 6 (54.5) |  |
| Triple-negative | 41 (31.3) | | 18 (43.9) | 23 (56.1) |  |

PDL-1, Programmed Death Ligand-1; CD8, Cluster of Differentiation 8. * P<0.05

**Table S3.** Hazard ratios for overall survival.

|  | OS | | | |
| --- | --- | --- | --- | --- |
|  | **Univariate analysis** | | **Multivariate analysis** | |
|  | **HR（95%CI）** | **P value** | **HR（95%CI）** | **P value** |
| Age |  |  |  |  |
| ≥50 vs. <50 | 2.399(0.912,6.311) | 0.076 | 2.922(1.073,7.953) | 0.036 |
| Tumor size (cm) |  |  |  |  |
| ＞2 vs. ≤2 | 1.333(0.24,2.846) | 0.458 |  |  |
| Nodal status |  |  |  |  |
| N2-3 vs. N0-1 | 2.843(1.344,6.014) | 0.006 | 2.458(1.150,5.256) | 0.020 |
| Pathological grade |  |  |  |  |
| Ⅲ vs. Ⅱ | 1.898(0.898,4.014) | 0.093 | 0.958(0.402,2.284) | 0.923 |
| PD-L1 |  |  |  |  |
| positive vs. negative | 0.921(0.425,1.995) | 0.834 |  |  |
| CD8 |  |  |  |  |
| positive vs. negative | 0.921(0.278,3.051) | 0.893 |  |  |
| Vascular invasion |  |  |  |  |
| Yes vs. No | 1.687(0.641,4.438) | 0.289 |  |  |
| Molecular subtype |  |  |  |  |
| TNBC vs. Not TNBC | 2.800(1.332,5.887) | 0.007 | 2.879(1.248,6.641) | 0.013 |
| KIF14 expression |  |  |  |  |
| high vs. low | 3.270(1.390,7.696) | 0.007 | 2.650(1.064,6.599) | 0.036 |

**Table S4.** Hazard ratios for recurrence-free survival.

|  | RFS | | | |
| --- | --- | --- | --- | --- |
|  | **Univariate analysis** | | **Multivariate analysis** | |
|  | **HR（95%CI）** | **P value** | **HR（95%CI）** | **P value** |
| Age |  |  |  |  |
| ≥50 vs. <50 | 1.265(0.607,2.633) | 0.531 |  |  |
| Tumor size (cm) |  |  |  |  |
| ＞2 vs. ≤2 | 1.164(0.596,2.273) | 0.658 |  |  |
| Nodal status |  |  |  |  |
| N2-3 vs. N0-1 | 4.650(2.309,9.364) | <0.001 | 4.118(1.988,8.486) | <0.001 |
| Pathological grade |  |  |  |  |
| Ⅲ vs. Ⅱ | 2.214(1.138,4.308) | 0.019 | 1.677(0.809,3.476) | 0.164 |
| PDL-1 |  |  |  |  |
| positive vs. negative | 1.127(0.552,2.300) | 0.744 |  |  |
| CD8 |  |  |  |  |
| positive vs. negative | 0.925(0.326,2.621) | 0.883 |  |  |
| Vascular invasion |  |  |  |  |
| Yes vs. No | 2.230(0.974,5.107) | 0.058 | 1.130(0.466,2.742) | 0.787 |
| Molecular subtype |  |  |  |  |
| TNBC vs. Not TNBC | 1.902(0.974,3.716) | 0.060 | 1.328(0.654,2.698) | 0.433 |
| KIF14 expression |  |  |  |  |
| high vs. low | 2.151(1.069,4.327) | 0.032 | 1.692(0.801,3.576) | 0.168 |
